# A molecular phylogeny of Hypnales (Bryophyta) inferred from ITS2 sequence-structure data

**DOI:** 10.1186/1756-0500-3-320

**Published:** 2010-11-25

**Authors:** Benjamin Merget, Matthias Wolf

**Affiliations:** 1 Department of Bioinformatics, Biocenter, University of Würzburg, Am Hubland, 97074 Würzburg, Germany

## Abstract

**Background:**

Hypnales comprise over 50% of all pleurocarpous mosses. They provide a young radiation complicating phylogenetic analyses. To resolve the hypnalean phylogeny, it is necessary to use a phylogenetic marker providing highly variable features to resolve species on the one hand and conserved features enabling a backbone analysis on the other. Therefore we used highly variable internal transcribed spacer 2 (ITS2) sequences and conserved secondary structures, as deposited with the ITS2 Database, simultaneously.

**Findings:**

We built an accurate and in parts robustly resolved large scale phylogeny for 1,634 currently available hypnalean ITS2 sequence-structure pairs.

**Conclusions:**

Profile Neighbor-Joining revealed a possible hypnalean backbone, indicating that most of the hypnalean taxa classified as different moss families are polyphyletic assemblages awaiting taxonomic changes.

## Background

Pleurocarpous mosses, which are mainly found in tropical forests, account for more than 50% of all moss species [1,2]. Brotherus in 1925 used morphological characters to partition the pleurocarpous into three orders. These were Leucodontales (= Isobryales), Hookeriales and Hypnobryales (= Hypnales) [3]. Later molecular analyses showed that the order Leucodontales, which was mainly defined by reduced peristomes, is polyphyletic due to convergent evolution [1,4]. The current concept divides the pleurocarpous mosses into: (1) the subclass Hypnidae (consisting of (i) The Ptychomniales with roughly 100 species, (ii) the Hookeriales with roughly 750 species and (iii) the Hypnobryales or Hypnales containing about 4,400 species), (2) the hypnodendroid pleurocarps (consisting of Hypnodendraceae, Racopilaceae, Cyrtopodaceae, Pterobryellaceae and Spiridentaceae) and (3) the rhizogonian mosses (Rhizogoniaceae, as well as several other species). The hypnodendroid pleurocarps and rhizogonian mosses, however, also include non-pleurocarpous mosses [2,5,6]. Hypnales comprise over 50% of all pleurocarpous mosses. This study analyzed the internal transcribed spacer 2 (ITS2) sequence-structure pairs from 35 hypnalean families encompassing a total of 1,634 species in order to test the hypothesis that the ITS2 sequence-structure can be used to determine the phylogeny of Hypnales and to resolve especially its phylogenetic backbone. A rapid radiation in the early history of pleurocarpous mosses has resulted in low molecular diversity generally, but particularly in the order Hypnales [5,7]. This has complicated the phylogenetic inferences from DNA sequence data due to the small evolutionary distances generated by phylogenetic signals [8]. The more variable the DNA marker, therefore, the better the resolution for low level phylogenies. The ITS2 is a spacer region between two conserved core genes, 5.8 S and 28 S ribosomal DNA. Since it does not code for a core gene, the ITS2 tolerates a higher mutation rate, which results in a more variable DNA sequence. The ITS2 sequence has been used to resolve moss phylogenies at the genus or species level [9,10]. But while the ITS2 DNA sequence is poorly conserved, the ITS2 secondary structure is strongly conserved, consisting of four helices with the third being the longest [11]. This conserved aspect of the ITS2 region has been exploited by several researchers to reveal deep phylogenies [2,12-14]. Two new computer programs, 4SALE [15,16] and ProfDistS [17-20] use both the variable and conserved characteristics of the ITS2 and in the process make the ITS2 the marker of choice for shallow and deep phylogeny analyses [21,22]. These programs allow the user to generate multiple sequence-structure alignments and Profile Neighbor-Joining trees using sequence and structure data simultaneously. Programs 4SALE and ProfDistS were therefore used in this study to generate a large scale phylogenetic analysis of 1,634 complete and correctly annotated hypnalean ITS2 sequence-structure pairs, which were currently available at the ITS2 Database [11-14,23,24].

## Phylogeny of Hypnales

### Large scale approach

**Figure 1:**
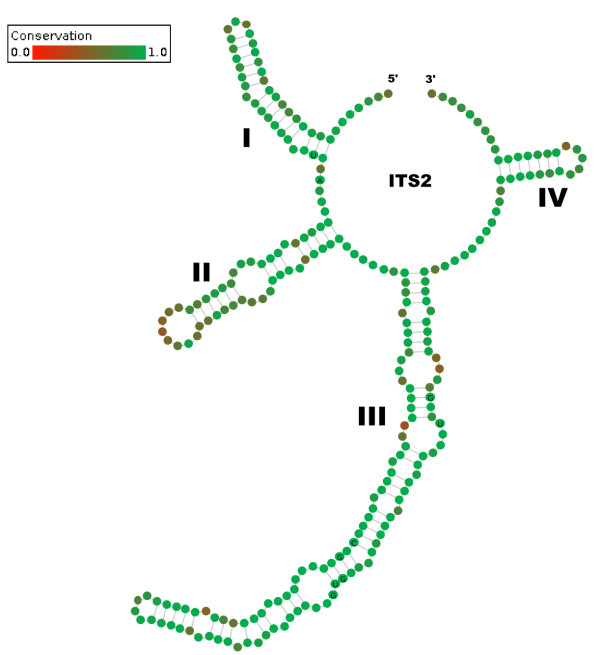
**51% consensus ITS2 secondary structure**. The four helices are numerated from I to IV. 100% conserved positions are indicated by an A, G, T or U within the green node. Each single bond is 90% conserved over the whole alignment.

**Figure 2:**
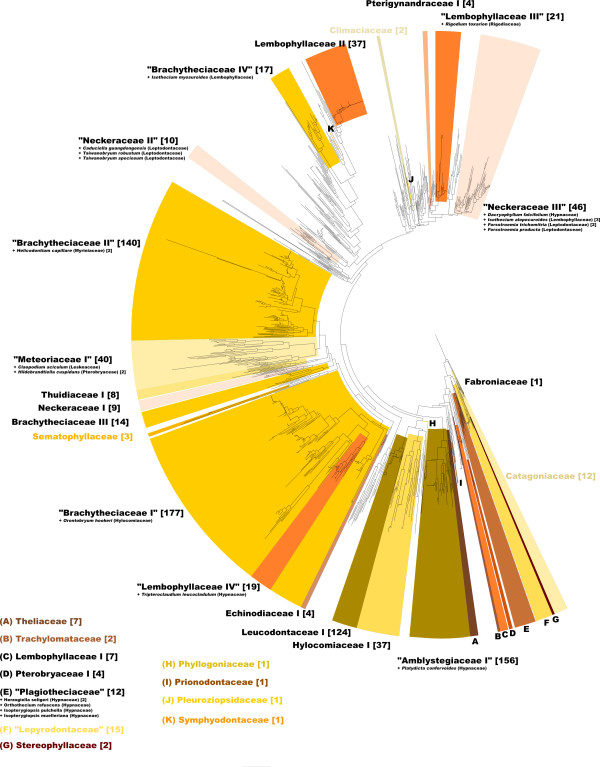
**Neighbor-Joining tree of 1,634 hypnalean sequence-structure pairs**. Mono- and paraphyletic clades are highlighted and labeled. Paraphyletic clades are indicated by quotes. A colored label denotes a clade as a complete monophylum containing each used sequence of this family. It is possible that some clades are labeled with "I", although there is no corresponding clade "II". In this case the missing sequences are spread in the non-monophyletic (uncolored) areas of the tree. Numbers in squared parentheses show the quantities of sequences in the specific clade. An apparently small clade can yet contain many sequences. In this case different sequence-structures of the same species built a monophylum and were collapsed for reasons of clarity and comprehensibility. Collapsed clades are marked by triangles instead of lines within the tree.

**Figure 3:**
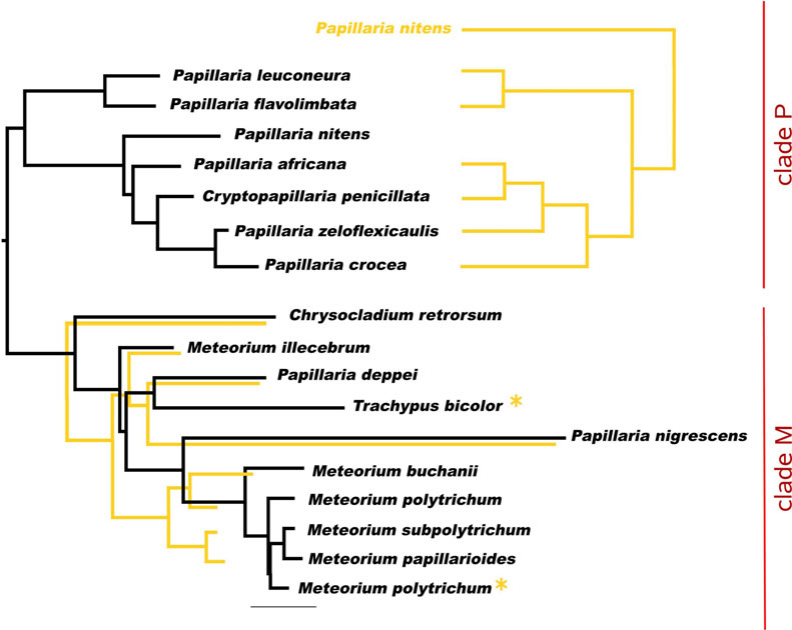
**Zoom in "Meteoriaceae I"**. Beige asterisks mark sequences that are not available in Quandt et al. [25]. Beige branches depict the tree as shown in Quandt et al. [25].

The alignment and editing tool 4SALE [15,16] generated a 51% consensus ITS2 secondary structure from all 1,634 sequence-structure pairs. The ITS2 consensus secondary structure consisted of the expected conserved four helices with the third being the longest (Figure 1). Every single base pair is 90% conserved over the whole alignment. This is evidence for the correctness of the 4SALE [15,16] alignment of over 1,500 sequence-structure pairs. Furthermore it underlines the strong preservation of the ITS2 secondary structure which allowed us to proceed on this basis. Using an ITS2 specific rate matrix, we calculated a phylogenetic tree of all 1,634 sequence-structure pairs with ProfDistS [17-20] (Figure 2, Additional file 1, Figure S1). Twentyfour of the 35 examined hypnalean families could be grouped to 32 clades, 11 of the examined families could be grouped to monophyla. Zooming in, a part of the subtree of the clade "Meteoriaceae I" was exemplarily compared to the 50% majority rule consensus tree as shown in Quandt et al. [25] and below. Both trees yielded similar results, validating the large scale approach (Figure 3). Another proof of the validity of the large scale approach is the clade "Neckeraceae II", consisting of 13 taxa. This clade shows remarkable similarities to analyses of Olsson et al. [26]. Both analyses show a distinct separation into two clades each, in particular *Pinnatella *and *Taiwanobryum*.

**Figure 4:**
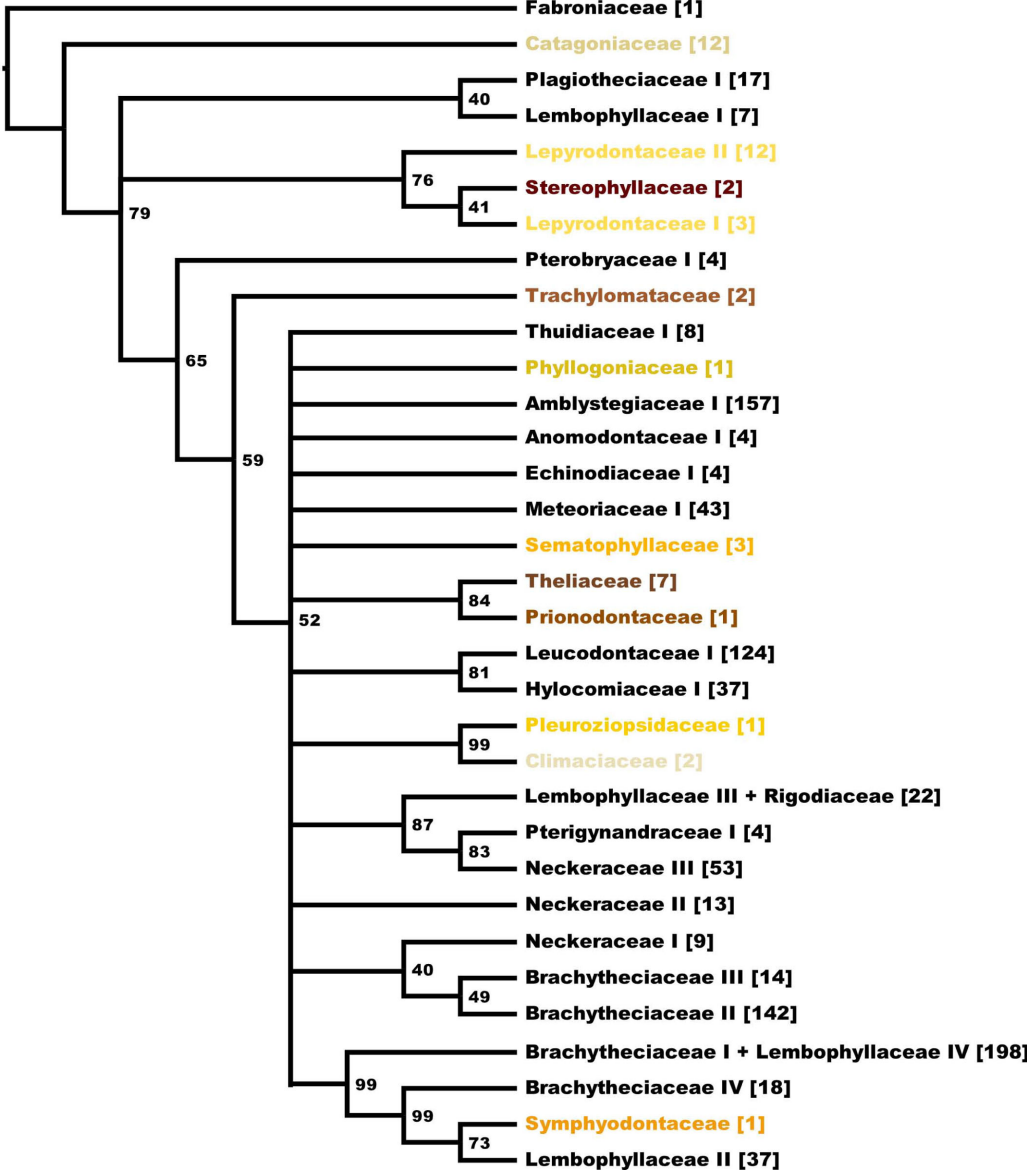
**Backbone of Hypnales**. Profile Neighbor-Joining with 100 bootstrap replicates. Bootstrap values of 40% and above are depicted. Clades with a bootstrap support of less than 40% are collapsed to a multifurcation.

After setting the highlighted clades in Figure 2 as predefined profiles for ProfDistS [17-20] with Cartoon2Profile a Profile Neighbor-Joining tree using 100 bootstrap replicates was computed. The remaining sequences lying in the non-monophyletic rests of the distance tree, were not considered to confine this computation to only the backbone of the Hypnales (see below), and how it is supported regarding the ITS2 sequence-structure alignment (Figure 4).

### Meteoriaceae

Quandt et al. [25] provided a detailed survey about the Meteoriaceae focusing on the genera *Meteorium *and *Papillaria*. A maximum likelihood tree of their research (hereinafter referred to as Meteoriaceae Q) is now to be compared to the subtree "Meteoriaceae I" (Figure 3). Both trees illustrate the same characteristic division into clade P and clade M. Internally clade M differs in solely a few details: The Meteoriaceae Q handle *Meteorium buchanii *and *Meteorium polytrichum *together as a sister to *Meteorium subpolytrichum *and *Meteorium papillarioides*. In "Meteoriaceae I", however, *Meteorium polytrichum *itself is a sister to *Meteorium subpolytrichum *and *Meteorium papillarioides*. The three together stand as a sister group to *Meteorium buchanii*. Furthermore *Papillaria nigrescens *and *Papillaria deppei *build a monophylum with *Meteorium illecebrum *as a sister. Within clade P of "Meteoriaceae I" *Papillaria crocea*, *Papillaria zeloflexicaulis*, *Cryptopapillaria penicillata *and *Papillaria africana *show an exactly reverse descent as in Meteoriaceae Q. Moreover, in Meteoriaceae Q *Papillaria nitens *is a sister to the complete remaining clade P and not only to the *Papillaria africana *clade. Nevertheless the facts that (1) the "Meteoriaceae I" strongly resemble published results [25] and (2) the ITS2 consensus secondary structure of all 1,634 sequence-structures shows the specific ITS2 characteristics, underlines that the ITS2 sequence-structure analyses were applicable to the analyzed taxonomic levels. A Neighbor-Joining analysis of the "Meteoriaceae I"-only alignment (also based on sequences and secondary structures simultaneously, data not shown) showed a few disagreements with the subtree "Meteoriaceae I" of the hypnalean NJ tree (Figure 2). Regarding clade M *Chrysocladium retrorsum *is no longer the only most basal taxon, but is joined by *Papillaria deppei*. Within clade P *Papillaria africana *is now directly related to *Papillaria nitens*.

### Backbone

Morphological convergence is widespread among the pleurocarps. This is one reason for the difficulties in moss systematics. Among the order of Hypnales several families (Hypnaceae, Neckeraceae, Leptodontaceae, Anomodontaceae, Hylocomiaceae, Amblystegiaceae, Brachytheciaceae, Lembophyllaceae, Meteoriaceae and Leskeaceae) were recently revealed as polyphyletic [9,27]. This discovery shows several matches with the distance tree as shown in Figure 2. The Anomodontaceae were indeed non-monophyletic. The Brachytheciaceae are divided into two major and two minor clades (without considering the few sequences spread in the unhighlighted areas). The two major clades consist of 198 and 142 sequence-structure pairs, respectively. Furthermore, the Lembophyllaceae split up into three clades. One of these three clades ("Lembophyllaceae III") contains the only Rigodiaceae (*Rigodium toxarion*) of our taxon sampling. These findings strongly reflect the results of Quandt et al. in 2009 [28]. Along the history of moss classification *Rigodium *was placed in several diffrent families. In 1909 Brotherus originally ranked *Rigodium *among the Brachytheciaceae [29], later in 1925 among the Lembopyllaceae [3]. Due to short leaf cells and the similarity to Thuidiaceae in its habit Robinson [30] returned *Rigodium *to the Brachytheciaceae, dispite the lack of paraphyllia and distinct leaf papillae [28]. Whereas Buck [31] counted *Rigodium *among the Thuidiaceae, Crum [32] constituted the Rigodiaceae, which were accepted by Zomlefer [28,33].

Considering the distance tree (Figure 2) and Olsson et al. [9] there is now no doubt about the polyphyly of Hypnaceae and Leskeaceae, which are widespread over the whole tree. Therefore it is possible for a clade to contain a single hypnacean or leskeacean sequence, either because this species actually belongs into this group or because of failures in sequencing and/or annotation. Most families with 12 sequences or less could successfully be clustered as a monophylum (Climaciaceae [2], Theliaceae [7], Trachylomataceae [2], Sematophyllaceae [3], Stereophyllaceae [2] and Catagoniaceae [12]). There is evidence for the Thuidiaceae being a non-monophyletic group [34]. Despite one large thuidiacean clade with 8 taxa, this assumption could be confirmed, for there were several thuidacean sequences which showed no immediate relation to the Thuidaceae as shown in Figure 2.

While the distance tree could reveal phylogenetic relationships within families, Profile Neighbor-Joining successfully allowed us to gain information about the backbone of the hypnalean phylogeny (Figure 4). The Priodantaceae and Theliaceae build a monophylum in 84% of the calculated bootstrap replicates. The monophylum Climaciaceae + Pleuroziopsidaceae (99%) could already be observed in the distance tree (ignoring seven sequence-structure pairs of the close-by non-monophyletic rest). Now the monophyly can be confirmed by high bootstrap values.

Moreover, there is no doubt that the "Brachytheciaceae IV" are a sister to Lembophyllaceae II + Symphyodontaceae with "Brachytheciaceae I" (including Lembophyllaceae II) being a sister to this clade. Although "Brachytheciaceae III" and "II" meet during the PNJ process, they are still only distantly related to "Brachytheciaceae I" and "IV" and thus are polyphyletic. Also the three Neckeraceae clades do not meet during the PNJ, which could be a hint for the actual polyphyly, as outlined by Olsson et al. [9]. Despite the Lepyrodontaceae could successfully be grouped as a monophylum with a bootstrap support of 76%, provided that the Stereophyllaceae were included, the clade shows the same characteristics as in the distance tree (Figure 2). The Stereophyllaceae split the Lepyrodontaceae into two subgroups, making them paraphyletic. The "Hylocomiaceae I" and "Leucodontaceae I" build a monophylum with a bootstrap support of 81%.

By rooting the phylogenetic trees at Fabroniaceae we use the same root as Ignatov et al. [35]. Unlike our large scale ITS2 sequence-structure approach, Igantov et al. [35] used a compilation of sequence data of several markers for 144 sequences. Nevertheless, several families show similar relations. The most basal clade O1 consisting of Fabroniaceae, Stereophyllaceae, Plagiotheciaceae and several Hypnaceae is similar to our trees (Figure 2). The Clade O1 is gradually followed by Thuidiaceae, Amblystegiaceae and Sematophyllaceae, appearing in the main clade M1 of Ignatov et al. [35]. The main clade M2 and the trees of our analyses (Figure 2) have several taxa in common (Calliergonaceae, Hylocomiaceae, Neckeraceae and Brachytheciaceae) and thus confirm the suitability of the ITS2 marker for large scale phylogeny.

### Taxonomic implications

To describe the clades of the hypnalean backbone (Figure 4) as monophyletic several clades need to experience taxonomic changes in further studies. The Lepyrodontaceae, which are paraphyletic in the distance tree and in the backbone tree as well, can become a monophylum, if the Stereophyllaceae are integrated. Also the Rigodiaceae, which are located within the "Lembophyllaceae III", could actually belong to this group making it monophyletic. Furthermore the "Lembophyllaceae IV" are integrated into the Brachytheciaceae I. Eventually, the remaining clades become monophyletic, when (1) all sequences within the unhighlighted rest of the distance tree (Figure 2) are not considered and (2) scattered sequences within a family are now belong to the particular one. With PNJ 963 of 1,634 sequence-structure pairs could be grouped to 30 monophyla.

## Conclusion

The large scale analysis of Hypnales (Bryophyta) inferred from internal transcribed spacer 2 sequence-structure data successfully accomplished one step towards the deciphering of the taxonomy of pleurocarpous mosses. The use of not only ITS2 sequences, but also structural data is a plausible method, for it provides the feature of calculating evolutionary distances both at the level of highly variable sequences and at the level of conserved structures. Although further studies are needed and asked for to confirm the phylogenetic backbone of the Hypnales, the results of this survey show evidence for the actual polyphyly of many classic pleurocarpous families, still awaiting taxonomic changes. Now it is desirable, that Bryologists revisit specific groups within Hypnales in the context of our large scale analysis. Back to back research considering morphological characters of mosses on a small scale contrasting our approach in mega-systematics is absolutely necessary in establishing a taxonomy of Hypnales. Furthermore, novel methods, like the transfer of the sequence-structure and profile approaches to a Maximum Likelihood algorithm, could provide a great leap towards the true hypnalean phylogeny in the future.

## Material and methods

### Taxon sampling

1,730 hypnalean ITS2-sequences including their secondary structures were retrieved from the ITS2 Database [11-13,23,24] (retrieved on 12-20-2009, Additional file 2). A first filter searched for sequences with ≥ 98% sequence identity, which are yet classified within different families regarding the NCBI taxonomy database [36,37]. Ninetytwo sequences were deleted. 1,638 ITS2 sequence-structure pairs remained for further processing.

### Sequence-structure alignment and phylogenetic analyses

An automatic multiple sequence-structure alignment (MSA) was calculated with ClustalW [38] as implemented in 4SALE (A tool for Synchronous RNA Sequence and Secondary Structure Alignment and Editing) [15,16]. 4SALE provides the unique function of aligning sequences while simultaneously considering the secondary structure of each sequence. Using an ITS2 specific similarity matrix [15] 4SALE translates the sequence-structures into a mock protein with an alphabet of 12 letters (4 nucleotides multiplied by 3 possible states of being involved in the secondary structure [pair opening, pair closing, unpaired]). Based on the MSA a single distance based tree was calculated by ProfDistS [17-20] using an ITS2 specific General Time Reversible Substitution Model (GTR) [20]. Like 4SALE ProfDistS also considers the secondary structures of the used sequences. Now a second filter used a perl script which calculates the mean of all branch lengths and dismisses every sequence and every two-sequences-clade that is above a variable threshold (perl script available on request). The threshold was set to 50, which means that each sequence and two-sequences-clade with a branch length higher than 50 multiplied by the mean of all branch lengths was removed. Four sequences were classified as artifacts and deleted. The MSA, distance- and tree calculation were repeated with the filtered set of sequences, now containing 1,634 sequence-structure pairs (Additional file 3). Due to the large number of sequence-structure pairs, we took advantage of the Profile Neighbor-Joining (PNJ) function of ProfDistS. Clades obtained from the distance tree corresponding to the classic hypnalean taxonomy and containing at least 10 sequences were chosen as profiles. Moreover clades were converted into profiles if they contained less than 10 sequences, but still the majority of all sequences of a particular family, e.g. Sematophyllaceae, with only three sequences. By using 33 predefined profiles, it was possible to perform a full PNJ analysis using 100 bootstrap replicates. During each iteration of ProfDistS (1) nodes with a bootstrap value of 75 or more and (2) nodes containing sequences that are 90% identical are clustered as an additional profile. Thus it is possible that a node which was below the threshold during the last iteration now slides above the threshold, resulting in a super-profile. This process stops when no new profiles can be built. ProfDistS used an ITS2 specific rate matrix for the distance calculation [20]. A clade which is supposed to be a predefined profile can be marked as a cartoon in FigTree [39]. The perl script Cartoon2Profile http://profdist.bioapps.biozentrum.uni-wuerzburg.de/cgi-bin/index.php?section=cart2prof searches for every cartoon in a FigTree NEXUS file and converts the information into a ProfDistS compatible profile file.

A further sequence-structure alignment was generated containing only clade M and P of the "Meteoriaceae I". The alignment was processed by ProfDistS [17-20] using the ITS2 specific GTR [20].

All tree files (including the large distance tree, Additional file 4) were visualized with FigTree [39]. All figures were further processed with Inkscape [40].

## Competing interests

The authors declare that they have no competing interests.

## Authors' contributions

BM carried out the phylogenetic studies and drafted the manuscript under supervision of MW. MW conceived of the study and participated in drafting the manuscript. All authors read and approved the final manuscript.

## Supplementary Material

Additional file 1 **Figure S1**. The large distance tree (Figure 2) as a high quality png image. Click here for file

Additional file 2 **Used sequence-structure pairs**. Hypnales.xfasta contains 1,730 hypnalean sequence-structure pairs. Click here for file

Additional file 3 **Used sequence-structure pairs (filtered)**. Hypnales_filtered.xfasta contains the filtered set of sequence-structure pairs used for the final analyses including 1,634 sequence-structure pairs. Click here for file

Additional file 4 **Distance tree file**. The large distance tree (Figure 2) as tree file in Newick format. Click here for file
